# The Importance of Perceptual Experience in the Esthetic Appreciation of the Body

**DOI:** 10.1371/journal.pone.0081378

**Published:** 2013-12-04

**Authors:** Sonia Mele, Valentina Cazzato, Cosimo Urgesi

**Affiliations:** Dipartimento di Scienze Umane, Università di Udine, Udine, Italy; Istituto di Ricovero e Cura a Carattere Scientifico Eugenio Medea, San Vito al Tagliamento, Pordenone, Italy; University of Bologna, Italy

## Abstract

Several studies suggest that sociocultural models conveying extreme thinness as the widespread ideal of beauty exert an important influence on the perceptual and emotional representation of body image. The psychological mechanisms underlying such environmental influences, however, are unclear. Here, we utilized a perceptual adaptation paradigm to investigate how perceptual experience modulates body esthetic appreciation. We found that the liking judgments of round bodies increased or decreased after brief exposure to round or thin bodies, respectively. No change occurred in the liking judgments of thin bodies. The results suggest that perceptual experience may shape our esthetic appreciation to favor more familiar round body figures. Importantly, individuals with more deficits in interoceptive awareness were less prone to increase their liking ratings of round bodies after exposure, suggesting a specific risk factor for the susceptibility to the influence of the extreme thin vs. round body ideals of beauty portrayed by the media.

## Introduction

Body esthetics is a fundamental dimension of self-image that strongly impacts well-being and mental health. Importantly, esthetic body appreciation is influenced not only by the perception of body forms but also by the conceptions and ideals shared in a cultural environment. Several studies reported that an ideal of extreme thinness is conveyed by family, fashion and especially by mass media, particularly in rich and industrialized Western countries [1] [2]. The continuing presentation of ultra-thin models in the media leads people to internalize the beauty ideal of a lean body and contributes to increases in the degree of body dissatisfaction in adolescents and young adults [3]–[7]. The media exposure may play an important etiological role in the development of eating disorders (ED), which mostly affect women in rich and industrialized countries [8]. The ideal body is not only lean but it is also fit and muscular [9], [10]. However, the degree of body dissatisfaction is related to thinness rather than to fitness. Homan et al. [11] demonstrated a dissociation between the effects of exposure to normal-weight and athletic bodies and those of exposure to ultra-lean, athletic bodies and found only in the latter case a correlation with body dissatisfaction. So exposure to thinness causes negative feelings in relation to their own body [11]. What remains unclear, however, is the variable media exposure impact on individuals from the same society, contributing to the development of body image disorders and ED in only a subgroup of the society. This question points to a need for an in-depth exploration of the perceptual, cognitive and emotional processes involved in the effects of body exposure on esthetic body appreciation.

Three processes have been described in the experience-based reshaping of perception: perceptual priming, perceptual aftereffects, and norm-based coding. All these processes seem to rely on partially similar neural mechanisms characterized by an attenuation of the neural responses to the adapted stimulus features [12], but their involvement may lead to different predictions about the effects of perceptual adaptation on body esthetic appreciation. Perceptual priming reflects the facilitation of the perceptual coding of stimuli that have been previously encountered and has been found at many different levels of information processing according to the features of stimulus and task [13], [14]. The facilitation due to the physical attributes depends on the perceptual representation system and may be driven in a bottom-up manner [14]. According to priming effects, thus, exposure to thin body figures facilitates the perceptual processing of stimuli sharing the same features, likely biasing perception in the direction of the adapted feature (i.e., others' bodies are perceived as thinner than they actually are). Such thin-body priming effects may explain, for example, why people tend to pay more attention to thin bodies in particular or beautiful body parts in general [15], [16]. However, such attentional bias seems to have no relation with body dissatisfaction [16] and is reduced, rather than heightened, in patients with eating disorders [17], thus questioning its relation to the development of body image disturbances.

The perceptual aftereffects may often explain the socio-cultural influence on body image disturbances. The repeated exposure to stimuli with given features changes the subsequent perception in the direction opposite the adapted features [18]. These adaptation effects (or aftereffects) occur at many different levels of information processing, from low level visual features [19] to high level object and face representation [20], [21]. A change in the perceptual representation of bodies after repeated exposure to extreme thin bodies might, thus, explain the socio-cultural influence on body image disturbances, since exposure to thin bodies should make bodies to appear fatter. It is unclear, however, whether such perceptual changes may be linked to the long-term modifications of the esthetic judgments of thin vs. round human figures that characterize the current esthetic canons and the personal appreciation of one's own and other's body.

A possible mechanism to explain the influence of perceptual adaptation on the ideals of body beauty stems from norm-coding models of perceptual adaptation. In this view, the perception of the members of homogenous classes that share common configurations, such as faces and bodies, is based on the features of a template representation utilized as a reference point to perceive other exemplars[22]. The members that are more similar to the template receive higher esthetic appreciation[23]. Such norm-based representations may be shaped by experience [20], [21], thus favoring esthetic appreciation toward a preference for more familiar stimuli. Although the perceptual aftereffects and norm-based accounts attempt to explain the same pattern of findings, they call for different mechanisms involved in the experience dependents change of perception. Indeed, in the former, perception of stimuli is altered by adaptation but the internal criterion relative to which they are judged is not. Conversely, in the norm-based account, perception of stimuli changes as a result of the shift in the internal norm.

Studies of face attractiveness have documented that familiarity is a crucial factor that influences our esthetic appreciation of others' faces [24]–[26]. Indeed, people prefer average faces made from several photographs of male or female models as compared to any individual face and even to faces made from the fusion of only a few photographs [25], [26]. The average face may be preferred because we have a stronger feeling of familiarity with it, since any indications of individuality and uniqueness are dispersed [24]. Accordingly, recent studies have demonstrated that experience modulates the attractiveness judgments on faces [20] and our perceptions of what is normal or average in a face [27].

Fewer studies have, on the other hand, investigated how experience modulates body perception. These studies showed that exposure to both thin or round bodies modulates normality judgments, with a tendency to perceive the adapted figure as more normal [28]–[30]. Importantly, the effects of exposure to round bodies were less pronounced in individuals with higher body dissatisfaction and stronger internalization of Western ideals, supporting the connection between the effects of perceptual adaptation and the development of body image disturbances. However, in these previous studies [28]–[30] the same stimulus used during the exposure phase was presented for 6 s before each test image in the post-exposure evaluation phase. It is, thus, unclear whether the effects were due to a short-term perceptual contrast induced by the sequential presentation of the adapting and test bodies in each trial or to a long-term change of body normality beliefs and ideals after repeated exposure to extremely thin or round stimuli. Further lack of clarity questions whether the effects related to the perceptual representation of the bodies or affected bodily esthetic appreciation. Finally, the results indicate that the perceptual adaptation effects correlated with body dissatisfaction, but it is unclear which specific personality dimensions, which are typical among individuals with ED, may be associated with a heightened or reduced susceptibility to changing body esthetic judgments following the adaption to thin or round body models.

In the present study we investigated how the aesthetic judgment of the beauty of the body changes after exposure to different body weights and, in particular, whether such experience-dependent effects follow the prediction of the perceptual aftereffects or the norm-based accounts. Additionally we controlled if the effect of exposure was related to body dissatisfaction, internalization of Western ideals and personality dimensions associated with ED. We utilized a modified body adaptation paradigm that exposed participants to only extreme thin, only extreme round or a 1∶1 matched number of extreme thin and extreme round bodies for approximately 8 min. Before and after exposure, participants provided esthetic judgments on different male and female bodies of variable weight. We predict that in accordance with perceptual priming effects, the esthetic judgments of thin and round bodies should increase after their adaptation, with no effect on the non-adapted weight dimension. Conversely, following body size perceptual aftereffects, the test bodies should appear rounder after thin adaptation and thinner after round adaptation, biasing the esthetic appreciation toward more negative or more positive esthetic judgments, respectively. However, if body exposure changes the esthetic norms, esthetic appreciation should be biased toward the adapted weight dimension. Thus, liking judgments should become higher for thin bodies and lower for round bodies after thin exposure. The opposite should occur after round exposure. Crucially, we expect that the exposure effects relate to specific psychological dimensions that characterize the clinical profile of ED patients. Crucially, we expect that exposure effects should be related not only to body dissatisfaction but also to other psychological dimensions that characterize the clinical profile of ED patients and may mediate the particular susceptibility of these individuals to the effects of socio-cultural influence on body representation.

## Methods

### Ethics statement

The participants gave their written informed consent; the procedures were approved by the ethics committee of the Scientific Institute (IRCCS) “E. Medea” and complied with the ethical standards of the 1964 Declaration of Helsinki.

### Participants

Thirty-three students (18 female) of the University of Udine participated in the experiment and received course credit for their participation. They were 19–38 years of age (*mean* = 23.33, *sd* = 4.92) and reported normal or corrected to normal visual acuity in both eyes. The participants were naïve to the purpose of experiment and received information about the experimental hypothesis only after completion of the experimental tests. All but three participants were right-handed according to a standard handedness inventory [31].

### Stimuli

**Figure 1:**
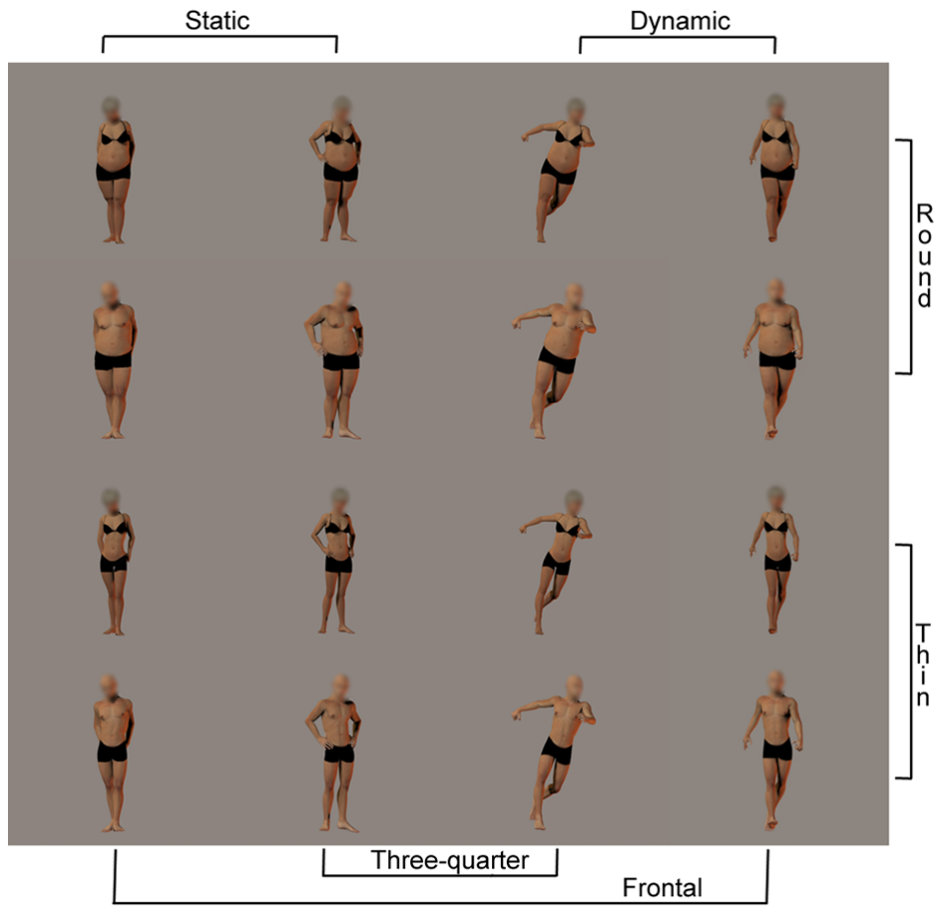
Body stimuli utilized in the exposure phases.

The stimuli were taken from a previous study [32] and depicted six 3-D human figure models (3 females) from the database of Poser Pro 2010 (e-frontier, Santa Cruz, CA). Each model was rendered in two static and two dynamic postures taken from a frontal or three-quarter view. For each posture, the models' body size was manipulated with the Poser software to have moderate to extreme levels of round and thin figures. A total of 16 images were created for each model. The models were depicted with the face scrambled, wearing black underwear and on a grey background to reduce the influence of non-bodily cues. The 16 images of four models (2 females) were utilized during the pre- and post-exposure evaluation phases (64 evaluation stimuli), whereas the extreme round and thin figure images of the remaining 2 models (1 female) were utilized for the exposure phase (16 exposure stimuli; Fig. 1). The body stimuli were used in a previous study in which we asked a large number of participants to judge the weight and other perceptual and affective dimensions of each stimulus [32]. The results of this study showed a parametric correspondence between the intended manipulation of body weight and the perceptual judgments of participants who rated the stimuli as varying from extremely thin to extremely round.

### Procedure

The experiment was composed of three daily sessions, each one consisting of three phases: i) initial evaluation of the stimuli (pre-exposure phase); ii) exposure phase; and iii) re-evaluation of the stimuli after exposure (post-exposure phase). The three sessions were conducted in three separate days with a waiting period ranging from three to seven days. The session order was balanced between participants. The participants in each session were administered the same pre- and post-evaluation procedures with different exposure conditions. In the two main exposure conditions they received only the 8 round body stimuli (round exposure) or the 8 thin body stimuli (thin exposure). In a third control exposure condition, participants received 8 round and 8 thin body stimuli, with a 1∶1 matching of the number of round and thin figures (control exposure).

During the experimental sessions, participants sat 57 cm away from a 15.6-inch LCD monitor (resolution: 1,024×768 pixels; refresh frequency: 60 Hz) on which stimuli appeared on a grey background and subtended a 10°×9° square region around the fovea. The stimulus-presentation timing and randomization were controlled with E-prime V1.2 (Psychology Software Tools Inc., Pittsburgh, PA) on a PC.

### Pre- and post-exposure phase

The 64 evaluation stimuli were randomly presented in three blocks for a total of 192 trials. The trials started with the presentation of a central fixation point lasting 500 ms, followed by the body image stimulus presented for 150 ms at the center of the screen. The experimenter monitored eye position by continuously checking the participant's gaze during the tachistoscopic presentation. The image persistence was limited by presentation of a random-dot mask (7.68°×7.68° in size; duration: 500 ms) obtained by scrambling the corresponding body stimulus with a custom-made image segmentation software. After the mask, the question “How much do you like it (*Quanto ti piace* in Italian)?” appeared on the screen with a vertical, 10-cm Visual Analogue Scale (VAS) ranging from “I like it very much (*Mi piace molto*)” (score = 100) to “I do not like it at all (*Non mi piace per niente*)” (score = 0). The top or bottom position of the two extremes was balanced between participants. The participants were asked to express an esthetic judgment on the body stimuli by moving the mouse cursor onto the point of the VAS corresponding to their opinion. The pre- and post-evaluation phase lasted approximately 10 min each.

### Exposure phase

The exposure stimuli were presented in three 48-trial blocks, with random presentation of male and female models, static and dynamic postures and front- and three-quarter-view body images, for a total of 144 stimuli. Each stimulus was presented for 1,000 ms and followed by a response frame that remained on the screen until response. The participants were asked to look carefully at the stimulus and respond immediately to one of the following questions presented after the offset of the stimuli in random order: “Male or female model (*Modello maschile o femminile*)?”, “Dynamic or static posture (*Postura statica o dinamica*)?” and “Front or three-quarter view (*Visione frontale o di mezzo profilo*)?”. The two alternative answers were displayed below the question. The subject's task was to press a button that spatially corresponded to the correct answer. The association between the answers and the buttons was balanced between participants. This procedure ensured that participants paid attention to the different morphological and dynamic aspects of the stimuli, limiting the cognitive load of the task after stimulus presentation. The exposure phase lasted about eight min.

### Psychological measures

At the end of the experiment, the participants completed three questionnaires: i) the Body Shape Questionnaire (BSQ-34, 1 scale; [33]) to assess the degree of body dissatisfaction; ii) the Eating Disorder Inventory-2 (EDI-2, 11; scales; [34]) to investigate the psychological and behavioral characteristics associated with eating disorders; and iii) the Sociocultural Attitudes Toward Appearance Questionnaire-3 (SATAQ-3; 4 scales; [35]) in its Italian translation [36] to assess the degree of mass media internalization of the models presented by the mass media.

### Data analysis

We calculated the individual mean VAS values for each condition in the evaluation phase (64 trials per cell). The preliminary analyses showed that participants preferred the opposite-gender models. However, the gender of the participants and of the models did not influence the effects of exposure and was not considered in further analyses. The data were entered into a three-way 2×3×2 repeated-measures Analysis of Variance (ANOVA) with time (pre- and post-exposure), exposure (round, thin and control), and weight (round, thin) as variables. All pair-wise comparisons were calculated with the Newman-Keuls post-hoc test. A significance threshold of *p*<0.05 was set for all statistical analyses. The data are reported as the *mean*±*standard error of the mean* (*sem*).

To estimate the esthetic judgment change (EJC) after exposure, we calculated the ratio between the post- and pre-exposure VAS values for each participant and exposure condition, thus allowing an estimate of the judgment change independently from the absolute scale used by the participants in rating the stimuli. The higher EJC values correspond to a greater change in esthetic judgment. The Pearson's *r* coefficient between the individual EJC values and scores on the clinical scales were calculated with a Bonferroni correction procedure to control for multiple correlations (16 correlations). We also tested whether body weight and age may affect participants' esthetic judgments of others' bodies and used the Pearson's r coefficient to test the correlations of the body mass index (BMI) and age of each participant with their absolute values of esthetic judgments before and after exposure and the estimates of EJC.

## Results

**Figure 2:**
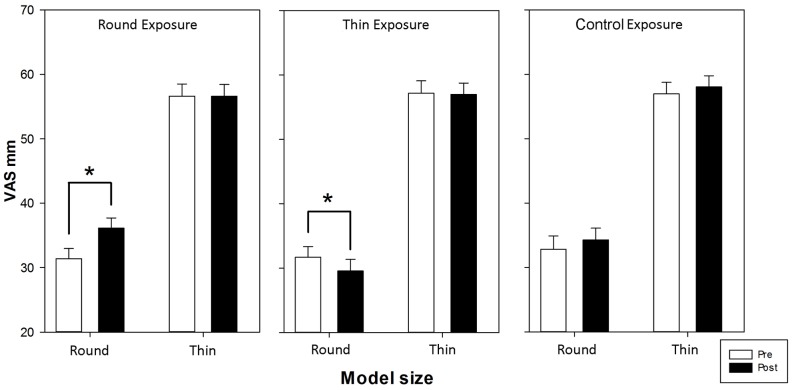
Mean (± *sem*) scores on the visual analogue scale (VAS) before and after the three exposure conditions. Asterisks indicate significant comparisons (*p*<0.05).

Figure 2 shows the esthetic VAS judgment values for round and thin model bodies before and after the three exposure conditions. The 3-way ANOVA revealed a significant main effect of weight (*F_1,32_* = 151.532, *p*<0.001, *η_p_^2^* = 0.825) with thin models (56.94 mm±1.63) preferred over round models (32.51 mm±1.62) and non-significant main effects of time (*F_1,32_* = 2.121, *p*>0.1, *η_p_^2^* = 0.062) and exposure (*F_2,64_* = 2.230, *p*>0.1, *η_p_^2^* = 0.065). The 2-way interactions time×exposure (*F_2,64_* = 7.911, *p*<0.001, *η_p_^2^* = 0.198) and weight×exposure (*F_2,64_* = 4.462, *p* = 0.015, *η_p_^2^* = 0.122) were significant and further qualified by a significant 3-way interaction (*F_2,64_* = 11.678, *p*<0.001, *η_p_^2^* = 0.267). The post-hoc analysis indicated that only the esthetic judgments of round models were modulated by exposure. In particular, the round models received higher VAS liking judgments after round exposure (post: 35.89±1.61) compared to baseline (pre: 31.18±1.58; *p*<0.001), and they received lower VAS liking judgments after thin exposure (post: 29.44±1.77) compared to baseline (pre: 31.60±1.60; *p* = 0.009). The VAS esthetic judgments for thin models were not modulated by round (pre: 56.52±1.85; post: 56.39±1.83; *p* = 0.855) or thin exposures (pre: 57.18±1.90; post: 56.88±1.75; *p* = 0.665). No changes were observed after the control exposure for either round or thin models, and no differences were obtained between the baseline values for the three exposure conditions (all *p*s>0.074). The results in the post-exposure phase indicate no difference for thin models; conversely, the VAS esthetic judgments for round models were higher after round exposure than after thin (*p*<0.001) and control (*p* = 0.017) exposures and lower after thin exposure than after control exposure (*p*<0.001).

**Figure 3:**
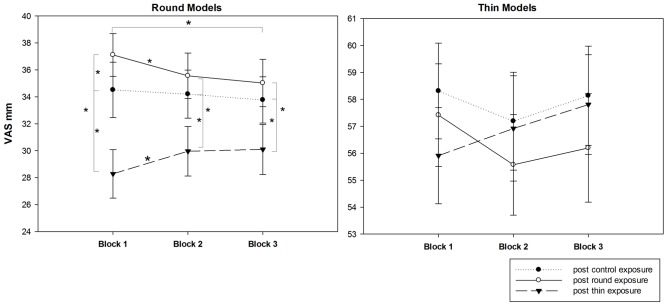
Mean (± *sem*) scores on the visual analogue scale (VAS) in the three successive blocks of the post-exposure phases. Asterisks indicate significant comparisons (*p*<0.05).

To test the duration of the change of the esthetic appreciation of round bodies, a post-hoc analysis was performed to separately compare the round and thin model judgments given in the three successive blocks of the post-exposure evaluating procedure (Fig. 3). The 2-way (block×exposure) repeated-measure ANOVA on the round body VAS judgments showed no significant main effect of block (*F_2,64_*<1) but significant effects of exposure (*F_2,64_* = 23.474, *p*<0.0001, *η_p_^2^* = 0.423) and interaction (*F_4,128_* = 3.558, *p* = 0.009, *η_p_^2^* = 0.1). Pair-wise comparisons showed that in the first block the round bodies received higher VAS ratings after round exposure (*p* = 0.006) and lower VAS ratings after thin exposure (*p*<0.001) when compared with the VAS ratings received after control exposure. However, the ratings of round bodies in the second and third blocks after round exposure were significantly lower than the first block (all *p*s<0.05) and not significantly different from the result after control exposure (all *p*s>0.38). Similarly, the VAS ratings of round models after thin exposure slightly increased from the first to the second block (*p* = 0.034) and remained constant from the second to the third block (*p* = 0.868). One important finding, however, indicates that the second and third blocks round bodies received lower VAS ratings after thin exposure than after control and round exposures (all *p*s<0.001). No significant effects were obtained from the ANOVA on the thin body ratings (all *F*<2, *p*>0.09). This result suggests that the esthetic judgment changes induced by round exposure rapidly faded away, but the effects of thin exposure remained constant during the 10-min duration of the evaluation procedure.

**Figure 4:**
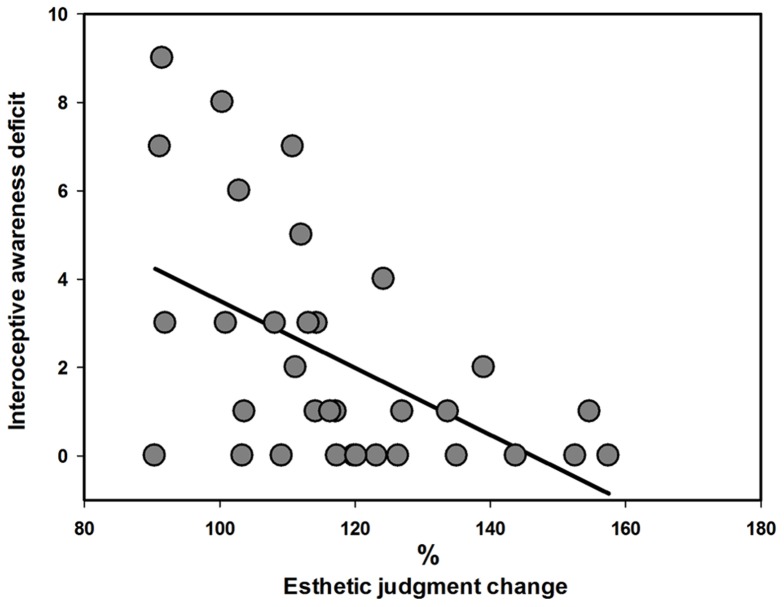
Correlation between priming effect in round condition for round models and the Interoceptive Awareness subscale of the Eating Disorder Inventory (EDI-2).

The correlation analysis showed that only the EDI-2 Interoceptive Awareness subscale was negatively correlated with the EJC after round exposure (*r* = −0.519, corrected *p*  = 0.031), whereas the other variables did not predict the amount of EJC (−0.39<*r*<0.27). Thus, individuals with higher interoceptive difficulties showed less increase of round body esthetic appreciation after exposure to round models (Fig. 4). No significant correlation was found between the BMI and age of the participants and their liking judgments in the pre- and post-exposure sessions as well as EJC (−0.23<*r*<0.32; *p*>0.075).

## Discussion

The present study aimed to investigate the effects of brief exposures to extreme weight figures on the esthetic appreciation of the human body. The results showed that exposures to round or thin figures exert an opposite modulation on the esthetic judgments of round models only. The strong preference for thin bodies was not altered after round exposure; thin bodies received largely higher liking judgments than round bodies in all conditions. Nevertheless, a very brief exposure lasting just 8 min mitigated the strongly polarized preference for thin bodies. After controlling for other factors that may affect real life body esthetic perception, our manipulation of perceptual experience may simulate the processes involved in the socio-cultural influence on the establishment of body esthetic ideals.

The esthetic rating changes cannot be explained by any non-specific effects of evaluation repetition for several sessions. The opposite modulations were observed after round and thin exposures, and no modifications were observed in the control condition during which participants repeated the task after exposure to both thin and round body models. On the other hand, the effects of extreme weight adaptation, particularly round exposure, tended to diminish during the short 10-min evaluation period. This result suggests a rapid return to the initial state after a balanced presentation of round and thin bodies. This finding keeps with a reshaping of the body perceptual representation system that is driven by the perceptual adaptation to a given body weight [18].

Such modifications of body esthetic appreciation concur with previous studies that tested the effects of body weight adaptation on a perceived normal body weight [28]–[30]. The exposure to both thin and round bodies modulated body normality judgments, and participants tended to normalize the adapted weight. Our separate testing of how the esthetic judgments of thin and round bodies change after exposure to extreme body weight aims to disentangle which specific mechanisms (i.e., perceptual priming, perceptual aftereffects or norm-based coding) are involved in the experience-dependent reshaping of esthetic body perception.

The results showed a change of round, but not thin, body perception after the exposure to round or thin models, revealing an asymmetric modulation that cannot be explained by any perceptual account. Indeed, perceptual priming processes [14], [37] would predict an increase of the esthetic judgments of the adapted stimuli only and fail to explain the decrease of the appreciation of round bodies after thin exposure. On the other hand, perceptual after effects [18] would predict an overall change of size perception toward the opposite direction of the adapted ones, with both fat and slim bodies being liked more after round body exposure and less after thin body exposure. This prediction however is in contrast with the fact that esthetic judgments of thin bodies were not affected after any exposure conditions. In a similar vein, norm-coding models [22] would predict an opposite, but symmetric shift of the esthetic judgments favoring the adapted size at the expense of the other. Thus, neither perceptual aftereffects nor norm-based coding explain why the thin esthetic judgments were not affected after any exposure condition. Such asymmetric modulation is not likely due to the ceiling of the measure. Not only were the VAS ratings far from close to the scale extremes, but the esthetic appreciation of thin bodies also failed to show any decrease after the exposure to round bodies.

Although no perceptual account can explain the absence of change of thin body esthetic perception, the results may stem from the interaction between the perceptual aftereffects and the norm reshaping processes. Indeed, both accounts predict an increase in round body liking after round exposure (for their thinner appearance or similarity to the norm) and a reduction after thin exposure (for their rounder appearance or distinctiveness with respect to the norm). Yet, the two accounts predict opposite effects for thin bodies. Indeed, the after-round-exposure perceptual aftereffects predict an increase and norm-based coding predicts a decrease in liking judgments; conversely, the after-thin-exposure perceptual aftereffects predict a reduction and norm-based coding predicts an increase in liking judgments for thin bodies. Thus, the perceptual aftereffects and norm reshaping might have mutually reinforcing effects for round bodies and have opposite and mutually deleting effects for thin bodies. The relative involvement of these two processes may subtend the differential effects of exposure to body models in real life or the media.

Such interpretative attempt, however, does not explain why previous body weight adaptation studies [28], [29] found a symmetric modification of the judgments of which body is considered more normal. Indeed, these studies found that exposure to both thin and round bodies modulates the normality judgments, with a tendency to consider more normal the adapted weight, thus in keeping with the prediction of norm-based accounts. Importantly, when Winkler and Rhodes [28] asked their participants to make a judgment of attractiveness, rather than of normality, they found that the judgment of attractiveness was modulated only after exposure to thin, but not round, bodies. Thus, exposure to a given body weight modified in a symmetric way a perceptual judgment (normality), following the predictions of norm-based coding, but it affected asymmetrically a complex evaluative judgment (attractiveness).We note that esthetic appreciation is determined not only by the perceptual features of the stimuli but also by the emotional reactions to them [32], [38]. The effects of weight exposure on esthetic body appreciation are possibly mediated not only by the plasticity of the perceptual representation system but also by changes of the emotional response to the adapted stimulus dimension. Indeed, Winkler and Rhodes [28] showed that unlike normality the judgments of body attractiveness were modulated only after exposure to thin bodies. The exposure to a given body weight symmetrically modified the perceptual judgments of normality, following the expectation of norm-based coding, and asymmetrically complex evaluative judgments of attractiveness, most likely because attractiveness involves a sexual arousal evaluation in which thinness plays an important role [39]. Accordingly, we suggest that repeated exposure to a stimulus triggering an emotional response, such as a human body, may induce a change of the hedonic value (liking judgments) and psychophysiological responses (e.g., heart rate). Such effects may be dependent on the valence of the stimulus [40], possibly explaining the different timing of the effects of exposure to round and thin bodies.

An important limitation of our study is the absence of a control condition in which other objects are used as stimuli. Therefore, we cannot establish whether the effect of the asymmetric modulation of round and thin figures is specific for bodily stimuli or might also extend to faces or other non-bodily objects. A previous study has shown that exposure to a non body object (coke bottle altered in size to appear thinner or rounder) exerted a symmetric influence on the normality judgments of similar objects [29], with the adapted object figure being evaluated as more normal. While these effects were similar to those obtained with body stimuli, body dissatisfaction and internalization of the Western ideal body of the participants were related to the effects of body exposure and not to those of the object exposure. This suggests that the effects of body exposure may have a clinical relevance for the onset and maintenance of body image disturbances, while those of the exposure to other objects do not. A different question is related to what extent exposure to round or thin objects may have cross-categorical influence on the esthetic judgments of thin and round bodies. This question, however, was not addressed in this study, which was aimed at investigating the role of different perceptual processes in the effects of body exposure, and further investigations are needed to better understand the body specificity, and more generally the within-category specificity of the effects of perceptual experience on esthetic appreciation.

The involvement of exposure-related modifications of the emotional and perceptual representations in the effects of body exposure was additionally supported in this study by the finding of a correlation between the amount of esthetic rating changes after round exposure and the individuals' scores on the Interoceptive Awareness subscale of the EDI-2. This subscale evaluates the uncertainty and confusion in recognition of and response to one's own and others' emotional states and the uncertainty in identification of the sensations of hunger and satiety. We found evidence that individuals with less ability to read and understand the sensory signals coming from the body (interoceptive awareness) are more resistant to increasing the esthetic appreciation of round bodies after repeated presentation. Interoceptive awareness is related to the strength of the emotional responses at the subjective and psychophysiological levels [41], [42]. Thus, people with greater ability to read the changes of the psychophysiological responses to adapted round bodies may more easily change the esthetic (hedonic) value attributed to them. In contrast, people with interoceptive awareness deficits may have difficulties in updating the esthetic value attributed to familiar stimuli on the basis of a change in their psychophysiological responses to it.

Importantly, patients with ED present severe difficulties in appropriate recognition and response to certain emotional states and visceral feelings, suggesting that “interoceptive confusion” is a core deficit in ED [43]. Furthermore, studies have shown that interoceptive awareness modulates body representation, and greater interoceptive sensitivity correlates with a more efficient integration of multi-sensory body-percepts [44]. Our results add to previous knowledge suggesting that interoceptive awareness may also mediate the experience-dependent shaping of esthetic body appreciation. However the subscale of the EDI-2 has not much power to test participants' interoceptive awareness as compared to other measures of it that use for example the ability to count heart beats. Future experiments are, thus, needed to actually test interoceptive awareness and its relation with the esthetic judgment.

On the other hand, in contrast to our expectations, no relation was observed between the change of esthetic judgments after exposure and the individual level of body dissatisfaction and internalization of Western ideals. This might be due to the fact that we did not find any modulation of esthetic preference for the thin bodies, whereas body dissatisfaction and Western ideals are specifically related to thin, rather than round bodies. Multiple factors not addressed in this study may modulate the esthetic appreciation of the body and how individuals react to the repeated exposure to extreme thin and round bodies. Along with the psychological dimensions classically associated to ED, here we tested the possible role of the familiarity with the participant's own body, operationalized with their BMI and age. No relation was observed between the participants' BMI and age and the absolute levels of esthetic judgments either before or after the three exposure procedures and their relative changes. It is possible, however, that the limited variability of our participants' BMI and age might have not allowed us to disclose such effects. On the other hand, the relative homogeneity of our sample and the full within-subject nature of this study design were adept to test and disentangle between the predictions that three accounts of the effects of perceptual experience, namely perceptual priming, perceptual adaptation and norm-based coding, have on body esthetic appreciation. Any of these perceptual accounts was confirmed in isolation, but the need to consider the interaction between different perceptual processes and the intervention of other emotional variables was highlighted by our results.

In sum, we demonstrated that a mixture of perceptual and emotional representation mechanisms may explain the effects of body exposure on the esthetic appreciation of human bodies with a round figure. Future studies are needed to better investigate the relative contribution of perceptual and emotional dimensions in the experiential related shaping of body esthetic. Crucially, interoceptive awareness may represent an important risk factor for both the susceptibility to the influence of extreme thin vs. round body ideals of beauty conveyed by the media and the development of stable negative evaluation of round human figures.
